# Noncontact Diffuse Reflectance Spectroscopy of Synovial Fluid Samples for Rapid Identification of Infections

**DOI:** 10.1002/jbio.202400213

**Published:** 2024-09-04

**Authors:** Erin E. Drewke, Robert L. Brand, Caroline G. Geels, Hanna K. Jensen, Kevin Wong, Jarret D. Sanders, Narasimhan Rajaram

**Affiliations:** 1 Department of Biomedical Engineering, University of Arkansas, Fayetteville, Arkansas, USA; 2 College of Medicine, University of Arkansas for Medical Sciences, Fayetteville, Arkansas, USA; 3 Department of Surgery, University of Arkansas for Medical Sciences, Fayetteville, Arkansas, USA; 4 Department of Radiology, University of South Alabama, Mobile, Alabama, USA; 5 Washington Regional Medical Center, Fayetteville, Arkansas, USA

**Keywords:** diffuse reflectance spectroscopy, joint infection, scattering, synovial fluid

## Abstract

Severe joint infections, such as septic arthritis, require rapid diagnostic testing of the synovial fluid aspirated from joints level so that a surgical team can be assembled quickly. We present a diffuse reflectance spectroscopy (DRS) system for noncontact determination of infection. Using a light-tight syringe holder and fiber optic probe, diffusely reflected light from 475 to 655 nm was acquired from 18 patient samples through the wall of a syringe in a noncontact and sterile manner. We determined the reflectance ratios at two different wavelengths—*R*_490_/*R*_600_ and *R*_580_/*R*_600_ and found statistically significant differences (*p* < 0.05) in both ratios between the infected and noninfected groups. Critically, the *R*_490_/*R*_600_ and *R*_580_/*R*_600_ ratios were significantly correlated with clinical biomarkers—the white blood cell (WBC) and red blood cell (RBC) counts, respectively. This study demonstrates the potential of DRS as a rapid diagnostic tool for joint infections.

## 1 | Introduction

Septic arthritis (SA) is a bacterial infection of joints, such as knee, hips, or shoulder, and can become a serious medical emergency leading to systemic infection or irreversible damage in adults and children [1–3]. SA is typically diagnosed by drawing a small volume of joint fluid into a syringe and analyzing the sample in the laboratory for C reactive protein, white blood cell (WBC) count, and erythrocyte sedimentation rate (ESR). Typically, a WBC count of 50 000/μL is considered the threshold for immediate operative treatment [4]. The laboratory tests typically take 30 min to over an hour to complete; in the case of pediatric patients, who commonly require sedation to draw the joint fluid, this means that they wait for the laboratory result under anesthesia. For adults, the waiting period adds critical time as an operative team is usually not assembled before the results are complete, thus delaying the procedure by upwards of 2–3 h, and can increase the odds of severe destruction to the joint. To reduce the time spent under anesthesia and to expedite treatment, there is a need for rapid diagnostic testing that allows physicians to quickly determine the severity of a joint infection and to quickly prepare appropriate medications or surgical operations.

Currently, there are no approved point-of-care devices capable of quickly diagnosing SA directly at the patient’s bedside. However, optical technologies have emerged as promising candidates for such point-of-care applications. Among these technologies, diffuse reflectance spectroscopy (DRS) stands out as a well-established noninvasive method. DRS utilizes optical fibers to administer broadband light and capture the diffusely reflected light (ranging from 400 to 650 nm) from biological tissue. Using models of light–tissue interaction, which either analytically or empirically describe the propagation of photons through scattering and absorbing mediums, it is possible to quantify the diffusely reflected light. This enables the extraction of pertinent information concerning tissue scattering characteristics and prominent tissue absorbers [5–8]. We have used DRS in several cancer studies, with an eye toward clinical translation, for monitoring tumor response to therapy [9–12] and differentiating between indolent and aggressive tumors [13].

While synovial fluid lacks tissue-like properties, it is still turbid media and is an ideal candidate for analysis using DRS, unlike fluids like blood and urine. Noninfected joints typically produce clear synovial fluid with a pale-yellow hue, whereas infected samples exhibit increasing cloudiness due to the presence of bacteria, proteins, and WBCs. This cloudiness arises from the multiple scattering of light by fluid particles. Elevated WBC levels are likely to increase the scattering cross-section, creating discernible characteristics in the wavelength-dependent diffuse reflectance spectrum. Conversely, clear synovial fluid, characterized by its pale-yellow coloration, exhibits fewer scattering events and has a distinctive absorption profile characteristic of its yellowish coloration. In scenarios where synovial fluid samples are blood-contaminated, spectral analysis algorithms can effectively discern between scattering and blood absorption events. Thus, quantitative analysis of diffuse reflectance spectra holds promise as a point-of-care tool for the quick diagnosis of synovial fluid infections.

Previous work on optical analysis of synovial fluid samples has mostly utilized the high chemical specificity of Raman spectroscopy to distinguish different stages of osteoarthritis [14, 15]. These studies placed a small volume of the synovial fluid sample on a glass slide to acquire Raman spectra. Short-wave near infrared spectroscopy [16] has been tested, albeit with limited success, because the study primarily focused on hemoglobin absorption in blood samples and utilized the spectral interference of scattering due to WBCs on absorption spectra to determine a relationship with WBC count. Absorption spectroscopy of synovial fluid samples determined that absorption by hemoglobin was too strong to detect possible infections but left open the possibility that light scattering information, if quantified, could provide diagnostic utility [17].

To ensure sample sterility, the ideal technology must be capable of making measurements on the fluid samples in a noncontact manner at the point of care. These samples are only viable for a certain amount of time outside of their storage conditions and could possibly be compromised in an open-air environment. Once part of the sample is taken from its sterile environment, it cannot be used for another test. Most DRS systems and their accompanying inverse models for quantifying light–tissue interactions are based on direct contact between the sample and fiber optic probe. While noncontact lookup table-based models have been developed in the case of optical probes axially offset from the sample to enable rapid wide-field spectroscopy [18], these require the sample to be open to air.

Here, we describe the development of a DRS system that can acquire spectra from fluids contained in a syringe. We demonstrate that light transport from a fiber optic probe through a syringe wall and into a solution held within the syringe allows the acquisition of diffuse reflectance spectra that can accurately capture the functional optical information of the sample. By retaining the synovial fluid drawn from joints within a sterile syringe, we can ensure that there is no reduction in fluid sample available for standard laboratory testing. Our preliminary results from a pilot clinical study demonstrate that we can successfully acquire DRS spectra from patient fluid samples and differentiate between infected and noninfected samples.

## 2 | Materials and Methods

### 2.1 | Diffuse Reflectance Spectroscopy

**FIGURE 1 |:**
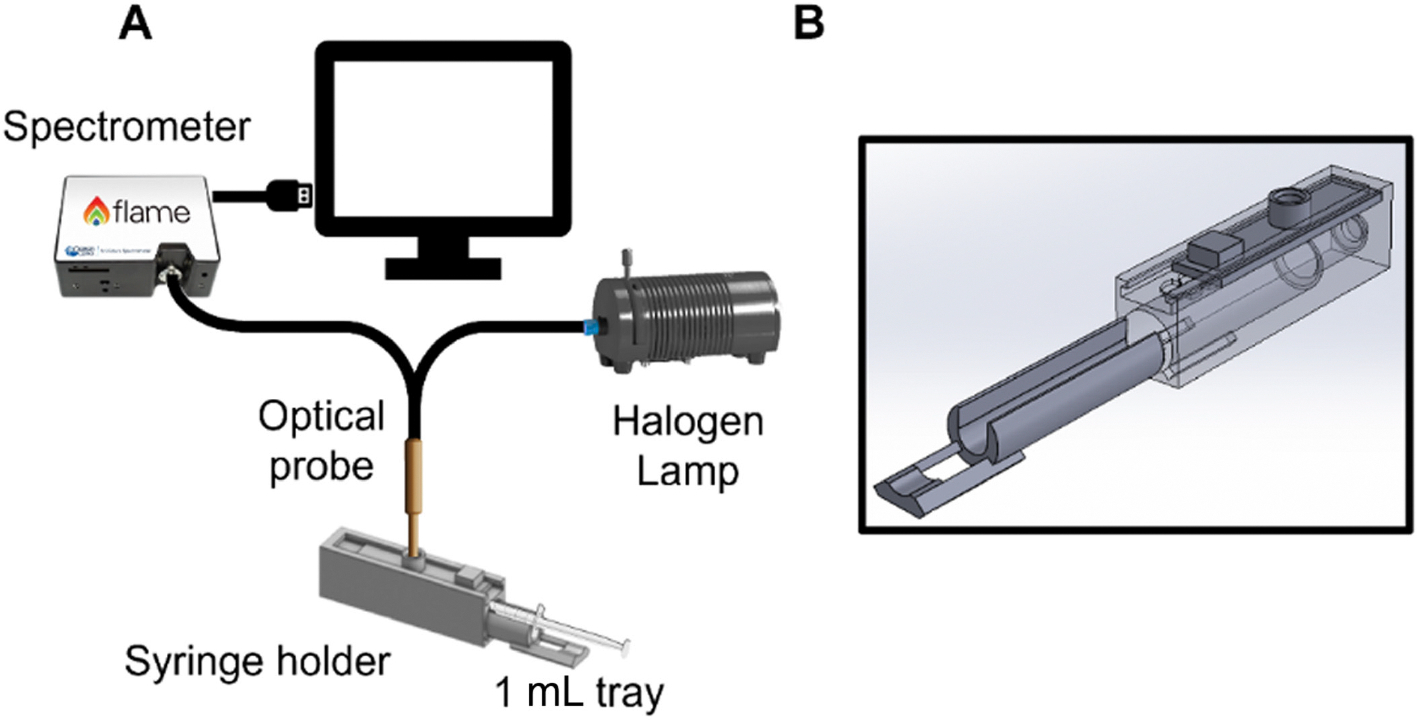
(A) Optical sensing platform. (B) Three-dimensional rendering of the syringe holder. The fiber optic probe is placed in the opening on the top. Depending on how much fluid is present, the location of the probe on syringe can be adjusted by sliding the top.

The system used to acquire optical spectra has been described previously [9, 13] and is shown in Figure 1. The system consists of a halogen lamp (HL-2000-FHSA-LL, Ocean Optics; Dunedin, FL) for source illumination, a USB spectrometer (Flame, Ocean Optics; Dunedin, FL) to collect the diffusely reflected light, and an optical probe for light delivery and collection. The probe consists of four illumination fibers arranged in a circle at the center of the sample end of the fiber optic probe and five detector fibers located at a source-detector separation distance (SDSD) of 2.25 mm. A custom-made syringe holder was designed using SolidWorks and 3D printed using black plastic filament. The inner diameter of the syringe holder was designed to securely fit a capped 10 mL syringe (without the needle). To accommodate patient samples with smaller volumes, a removable tray was designed, and 3D printed to fit 1 mL syringes. All synovial fluid drawn from patient joints was performed either using 10 or 1 mL syringes. The syringe holder was designed to ensure a tight fit for both 10 and 1 mL syringes and to block all ambient light. An opening at the top allows the introduction of the fiber optic probe into the holder to be placed in contact with the syringe. The probe opening is located on a slide to adjust the placement of the probe to three different locations on the syringe according to the fluid volume. This ensures that the probe is always measuring reflected light from a fully filled location within the syringe. Custom software written in LabVIEW (National Instruments, Austin, Texas) and MATLAB (MathWorks, Natick, MA) was used to acquire diffusely reflected light in the wavelength range of 475–655 nm with integration times ranging from 100 to 250 ms and lamp power ranging from 11.5 to 27.3 mW at 600 nm.

### 2.2 | DRS Spectra From Fluid Phantoms

**FIGURE 2 |:**
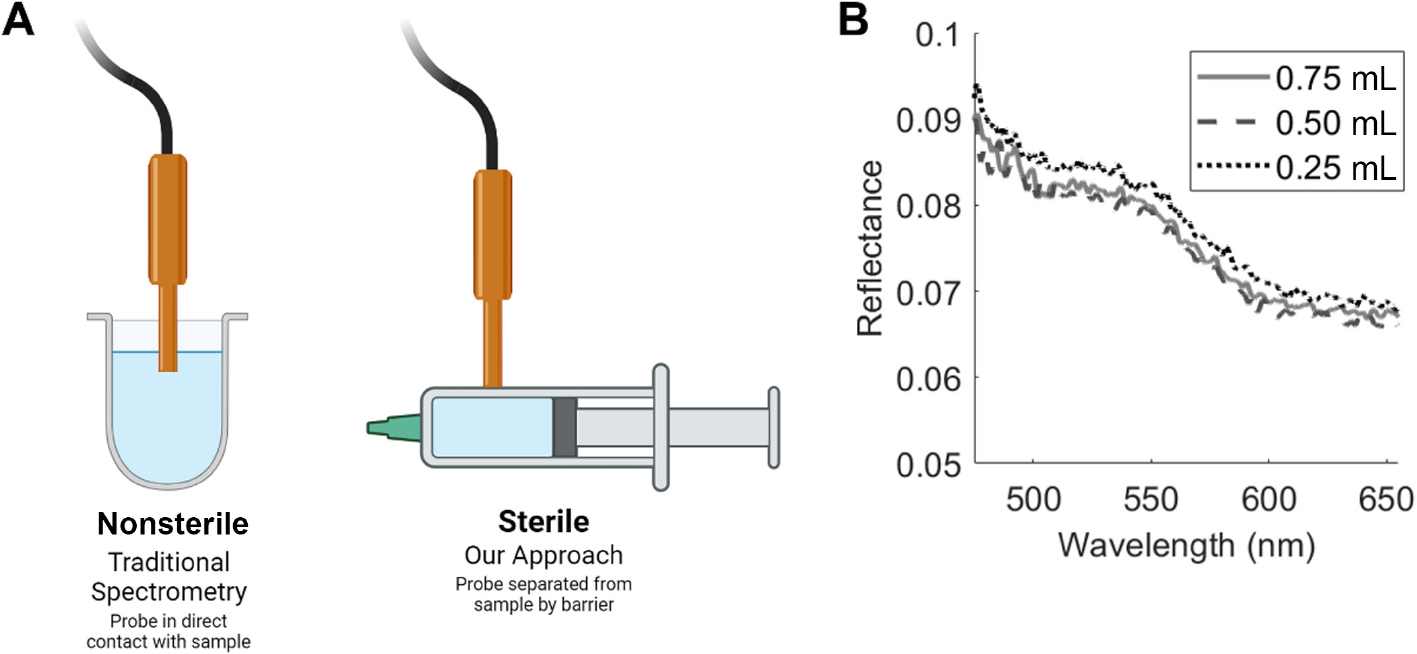
(A) Graphic showing a traditional DRS measurement when the probe is in contact with a solution and when it is being taken from outside of a syringe wall barrier (created with BioRender.com). (B) Diffuse reflectance spectra acquired from different volumes of a solution containing only polystyrene microspheres to simulate scattering (*μ*_s_ʹ(600 nm) = 2.1 cm^−1^). All three spectra were acquired with the solution placed in a 1 mL syringe and correspond to a total volume of 0.25, 0.5, and 0.75 mL.

To assess the feasibility of our approach, we created a series of fluid-mimicking phantoms with varying scattering and absorption properties and placed them in syringes for acquiring optical spectra. Using polystyrene microspheres (diameter = 0.75 μm; Polysciences, Warrington, PA) as the scattering medium, we fabricated eight phantoms with escalating levels of microspheres (1.4 × 10^8^, 2.29 × 10^8^, 4.57 × 10^8^, 8.58 × 10^8^, 25.73 × 10^8^, 34.31 × 10^8^, 45.74 × 10^8^, and 57.08 × 10^8^ parts/mL) suspended in water to simulate increasing levels of scattering. A separate set of phantoms was prepared with a fixed scattering level and three different concentrations of hemoglobin (Hb; 0.5, 1.0, 1.5 mg/mL). We made measurements with the probe in contact with the solution and after the fluid was aspirated into 1 mL syringes for measurement (Figure 2A). Acquired spectra were background subtracted to correct for ambient light and then divided by the background-subtracted light reflected from an 80% reflectance standard (Labsphere, North Sutton, NH) acquired at the same integration time and power settings to compute the diffuse reflectance.

### 2.3 | Patient Study

All studies were performed under an approved Institutional Review Board (IRB) protocol at the Washington Regional Medical Center (WRMC IRB, Form 1572). This study consisted of the collection and analysis of patient joint fluid draws at the WRMC Interventional Radiology Department. The data collected include the patient’s gender, age, the joint (shoulder/knee/hip) the sample is drawn from, the indication for the joint fluid sample, (suspected infection or other), and indication of rheumatoid arthritis, gout, or any other chronic condition that may affect the composition of the joint fluid. The sample lab report included information on the sample appearance, indication of solids/crystals, red blood cell (RBC) count, WBC count, and the percentage of cell types (lymphocytes, monocytes, basophils).

Synovial fluid was aspirated from the patient’s joint as per regular clinical protocol and transferred in a sterile manner to an appropriately sized syringe. The syringe was then placed in the syringe holder, and the slot for holding the fiber optic probe was adjusted so that spectra were acquired from a region of the syringe that was full. Three spectra were acquired from each sample. To calibrate daily measurements and compute the diffuse reflectance, optical spectra measurements were acquired from a 55% barium sulfate standard contained within a syringe of the same size as that of the patient sample.

## 3 | Results and Discussion

**FIGURE 3 |:**
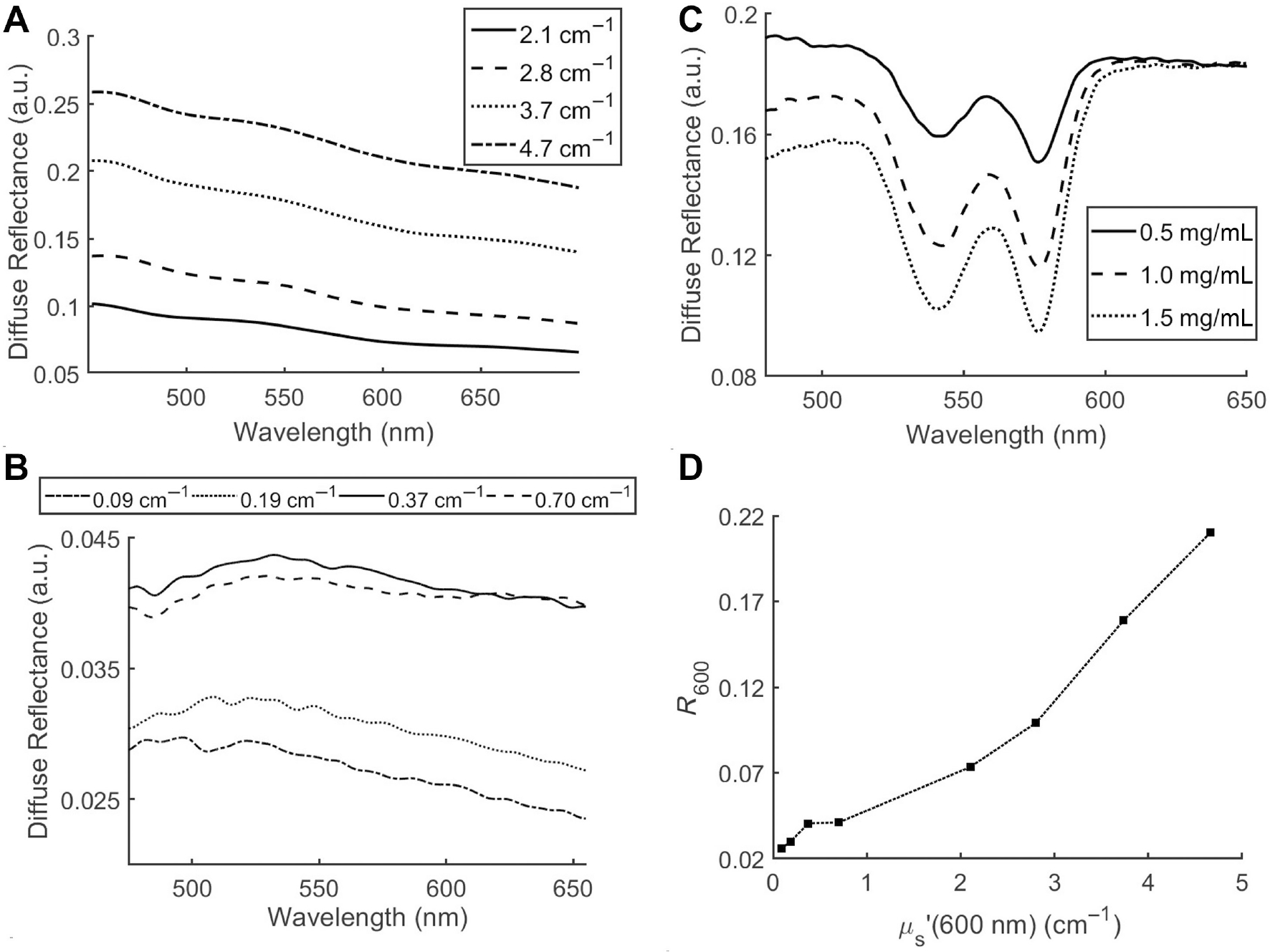
Diffuse reflectance spectra acquired from scattering-only phantoms in a 1 mL syringe at (A) high-scattering and (B) low-scattering levels. (C) DRS spectra acquired from phantoms containing a fixed level of scattering (*μ*_s_ʹ(600 nm) = 4.45 cm^−1^) and different concentrations of hemoglobin (in a 1 mL syringe). (D) Relationship between scattering and diffuse reflectance at 600 nm for the phantoms shown in (A) and (B).

We tested the ability of our probe to acquire DRS spectra from different fluid volumes. We prepared a scattering-only phantom containing polystyrene microspheres in water (25.73 × 10^8^ parts/mL corresponding to *μ*_s_ʹ(600 nm) = 2.1 cm^−1^). DRS spectra were acquired by filling the 1 mL syringe with different volumes of phantom—0.25, 0.5, and 0.75 mL. Spectra acquired for these different volumes are similar in line shape and intensity (Figure 2B). Figure 3A presents the diffuse reflectance spectra acquired from the four phantoms with the highest levels of scattering (2.1–4.7 cm^−1^ at 600 nm). We observe a power law dependence on wavelength that is similar to measurements obtained when the probe is in contact with the phantom (data not shown here). However, the four lowest scattering phantoms (*μ*_s_ʹ(600 nm) = 0.09–0.7 cm^−1^) do not demonstrate the same power law behavior consistently across all wavelengths (Figure 3B). Although we observe an increase in overall reflectance intensity with increasing scattering, wavelengths less than 550 nm demonstrate lower reflectance values, likely indicating an inability of the shorter wavelength photons at very low levels of scattering to reach the detector (located 2.25 mm away). Figure 3C presents phantoms with a fixed level of scattering (*μ*_s_ʹ(600 nm) = 4.45 cm^−1^) and increasing levels of absorption. These spectra clearly illustrate the changes in DRS spectra due to Hb absorption alone; the wavelengths longer than 600 nm corresponding to minimal absorption show no differences in DRS spectra, indicating the same scattering level across all phantoms. Figure 3D summarizes the relationship between reduced scattering coefficient and reflectance for the measurements performed in the syringe, indicating close to linear relationship between the two parameters for *μ*_s_ʹ(600 nm) > 2 cm^−1^. Overall, these measurements demonstrate the ability of DRS to acquire distinct optical spectra from phantoms of varying scattering and absorption levels through the wall of a syringe.

**FIGURE 4 |:**
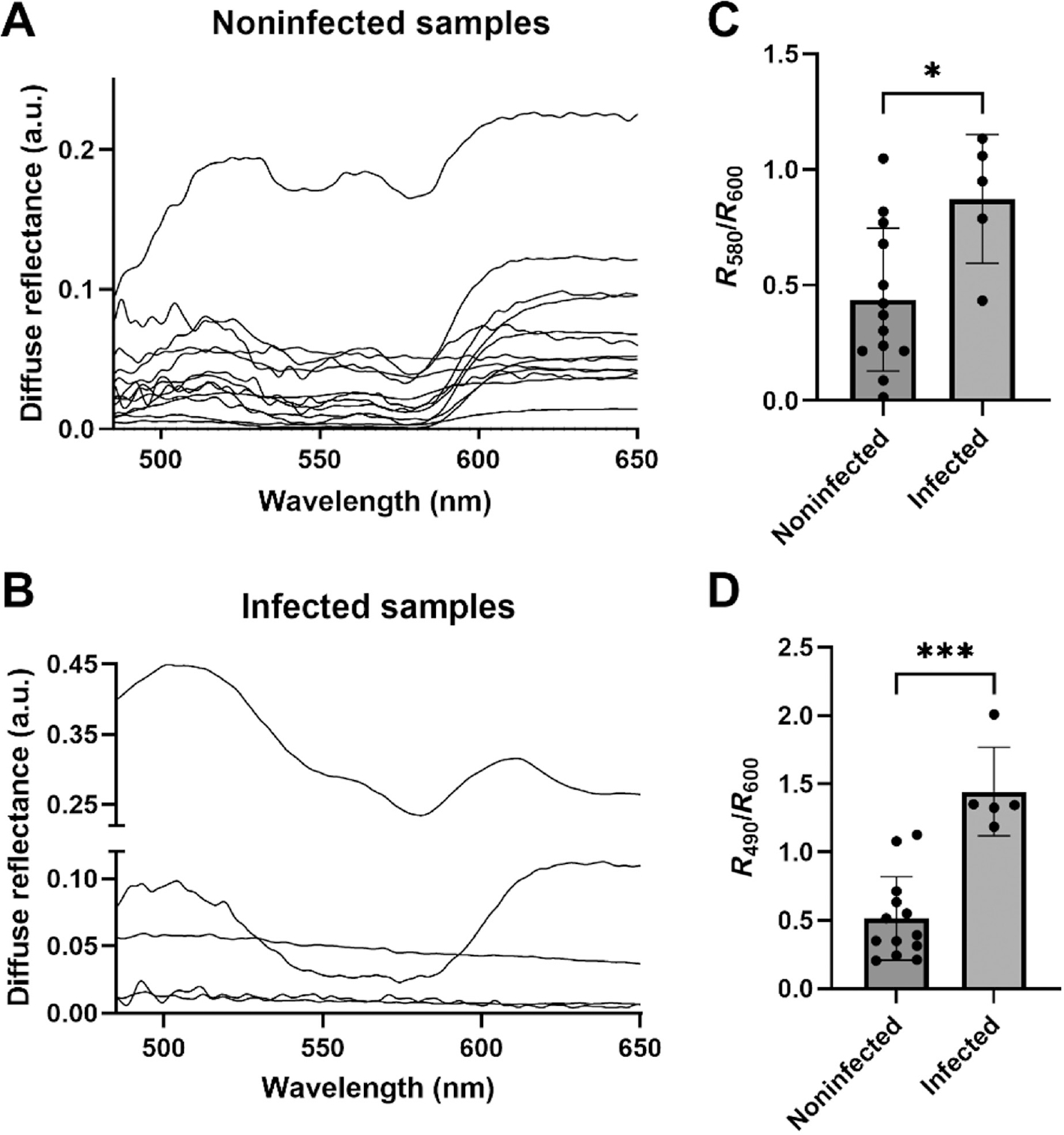
(A) DRS spectra acquired from noninfected patient synovial fluid samples (*n* = 13). (B) DRS spectra acquired from infected patient synovial fluid samples (*n* = 5). (C) A ratio of the reflectance at two wavelengths (580 and 600 nm) shows a statistically significant difference between noninfected (*n* = 13) and infected (*n* = 5) patient samples, *p < 0.05. (D) The ratio of *R*_490_/*R*_600_ is significantly different between noninfected (*n* = 13) and infected (*n* = 5) patient samples, *p < 0.05, ***p < 0.001.

Figure 4A,B presents DRS spectra acquired from infected (*n* = 5) and noninfected (*n* = 13) patient samples in the clinic. Out of the total of 18 samples, 11 were acquired from a 1 mL syringe and 7 from a 10 mL syringe. Several noninfected samples shown in Figure 4A have extremely low levels of reflectance. This can happen due to a couple of reasons—1. a high proportion of RBCs most likely due to a traumatic tap when obtaining fluid or when the fluid cavity had been otherwise compromised and 2. an injection of saline into nonnative joints to obtain better x-ray imaging contrast due to the presence of very little fluid. Further testing will be needed to determine the quality of the samples being measured and if it can be used for rapid diagnostics using DRS. Previous studies analyzing the accuracy of laboratory testing of synovial fluid WBC count have identified high RBC count and saline dilution as critical factors affecting sensitivity of laboratory testing [19].

To quantitatively analyze the reflectance spectra, we computed two reflectance ratios to represent scattering and absorption. We determined the ratio of reflectance at values at 490 and 600 nm (*R*_490_/*R*_600_) to represent the general shape of the scattering curve. We also calculated a ratio of reflectance values at 580 and 600 nm, corresponding to high Hb absorption and negligible Hb absorption, respectively. In addition to representing negligible absorption, the reflectance at 600 nm allows normalization of each reflectance sample and hence easier comparison across samples. The ratio of *R*_580_/*R*_600_ was significantly different (*p* < 0.05) between the infected and noninfected patient samples (Figure 4C). For the 13 noninfected samples, a majority had a ratio less than 0.5, indicating the presence of Hb in the samples, whereas the majority of the 5 infected samples had a ratio closer to 1, indicating negligible Hb absorption. Similarly, we found statistically significant differences (*p* < 0.001) in *R*_490_/*R*_600_, with infected samples showing a significantly lower ratio compared with noninfected samples (Figure 4D). A lower *R*_490_/*R*_600_ could be attributed to differences in scattering power, which can be influenced by scatter size [20, 21] and the effects of Hb absorption.

**FIGURE 5 |:**
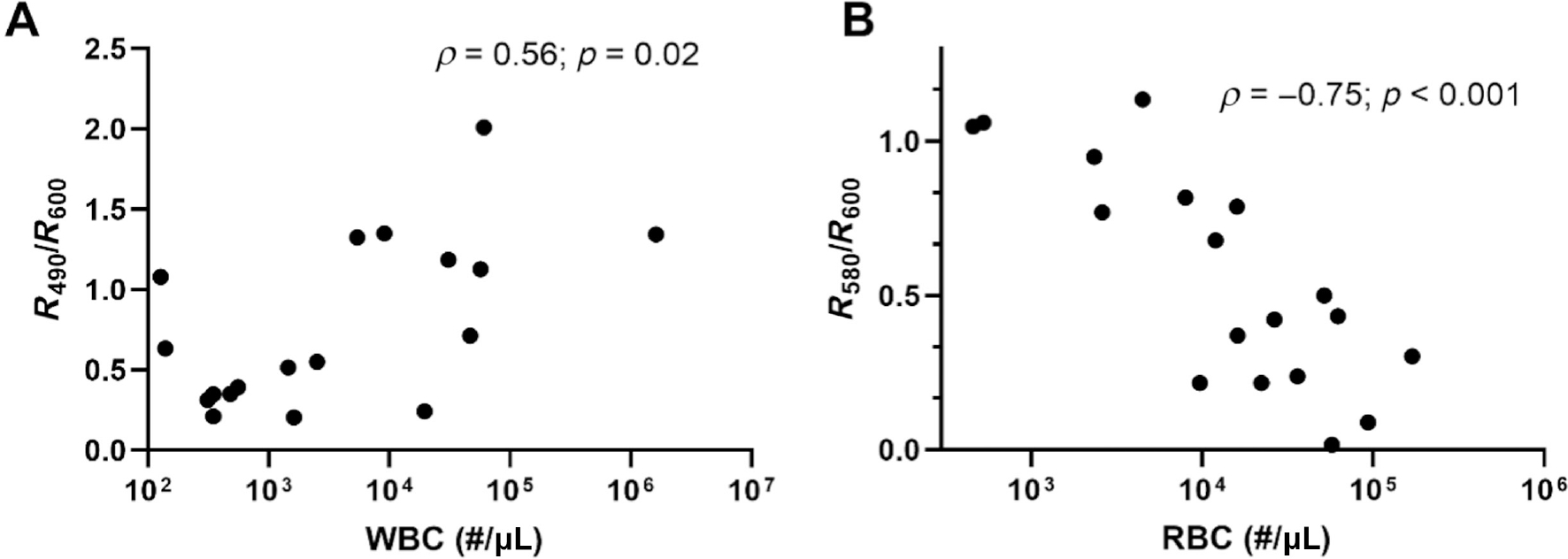
(A) The ratio of *R*_490_/*R*_600_ is positively correlated with WBC count. (B) The ratio of *R*_580_/*R*_600_, which represents Hb absorption, is negatively correlated with RBC count. For both correlations, Spearman correlation coefficient was calculated.

Our selection of the two reflectance ratios here were based on our understanding of the biological contributions of tissue to reflectance spectra and the typical setup of a light–tissue interaction model to quantify tissue optical properties. To validate this selection, we determined the relationship between the reflectance ratios and clinically determined WBC and RBC counts (Figure 5). Given the strong hemoglobin absorption at 580 nm, we evaluated the Spearman correlation coefficient between *R*_580_/*R*_600_ and RBC counts. We found a statistically significant negative correlation between the two parameters (*ρ* = −0.75; *p* < 0.001), demonstrating increased light absorption with increasing RBC counts. The *R*_580_/*R*_600_ ratio was not correlated with WBC counts (*ρ* = 0.28; *p* < 0.25). Similarly, we found a statistically significant positive correlation between *R*_490_/*R*_600_ and WBC counts (*ρ* = 0.56; *p* = 0.02), indicating higher reflectance values at shorter wavelengths for higher WBC counts. Again, the *R*_490_/*R*_600_ was not correlated with RBC counts (*ρ* = −0.43; *p* = 0.07).

As part of our ongoing pilot study, we are investigating different variables that could potentially impact the ability of our system to extract quantitative parameters that can be directly correlated with laboratory-based reports of fluid content. As we have observed in the patient samples already collected, the intensity range for the samples in some cases is significantly lower than the values that were measured in the phantoms. Our ability to accurately quantify these values will require modifications of the system such as changing the source-detector distance and the source light input. The study presented here used a SDSD for 2.25 mm. We are investigating whether shorter source-detector separations might be more sensitive to changes in the WBC count within these synovial fluid samples. Previous studies have shown that the linear relationship between scattering and reflectance breaks down at relatively longer SDSDs (~1.75 mm), enabling increased sensitivity to absorption and insensitivity to changes in scattering [22]. Another factor that could influence light delivery and collection is the syringe itself. The refractive index of the syringe material (1.49) is very close to that of the fiber core (1.46), this ensures that specular reflection at the boundary between the two media is minimal. While the inner diameter of 1 mL syringes is standard (~4.7 mm), we have found large variations in the syringe wall thickness (1.25–2.5 mm) depending on the manufacturer; this variation will impact light delivery and collection from the fiber. Detailed Monte Carlo simulations will be necessary to determine light travel within the syringe for different wall thicknesses and source-detector separations for the purpose of optimizing the system.

Outside of Monte Carlo-based models, testing will be needed to determine the signal to noise ratio of DRS spectra in response to different levels of controlled concentrations of WBC-like particles. While we were able to see differences in the initial studies using polystyrene microspheres with a diameter of 0.75 μm, further studies are required to account for the specific particle sizes that would be seen in a synovial fluid sample. Various models have been developed for characterizing leukocytes, ranging from simple single-layer spheroids to more complex five-layer structures [23]. The more intricate models consider factors like nucleus size and its influence on the overall scattering coefficient. Using a single-layer model enables physical representation of WBC concentrations with polystyrene beads [23–25].

In addition to using a simple ratio of two reflectance values, models of light–tissue interaction can be used to fit the DRS spectra and recover the optical properties of each patient sample. As discussed earlier, such models can be used only if samples are deemed to be free of any saline dilution that distorts the true scattering and hence WBC count within the sample. These models can also provide additional diagnostic information, such as the slope of the sample that could possibly better differentiate infected and noninfected samples.

## 4 | Conclusion

In summary, our preliminary testing demonstrates that DRS spectra can be acquired from sterile joint fluid samples held within a syringe with the optical probe placed on the outside of the syringe. Using scattering and absorption phantoms, we were able to see distinct changes when varying the amount of scattering and absorption concentrations. The analysis of patient synovial fluid samples shows that there are differences in the reflectance profiles of infected and noninfected samples, showing a potential for diagnostic value. Reflectance ratios corresponding to absorption and scattering within these samples were significantly correlated with RBC and WBC counts, respectively, demonstrating the translational potential of this application. However, further investigation is needed to account for specific markers in synovial fluid samples and to address challenges including sample quality due to saline dilution and RBC contamination. Our ongoing study aims to use whole spectrum analysis to increase the accuracy of our diagnostic approach, leading to improved rapid diagnosis of joint infection.

## Data Availability

The data associated with this paper will be made available on GitHub.
